# Single-cell spatiotemporal analysis reveals alveolar dendritic cell–T cell immunity hubs defending against pulmonary infection

**DOI:** 10.1038/s41421-024-00733-5

**Published:** 2024-10-16

**Authors:** Boyi Cong, Xuan Dong, Zongheng Yang, Pin Yu, Yangyang Chai, Jiaqi Liu, Meihan Zhang, Yupeng Zang, Jingmin Kang, Yu Feng, Yi Liu, Weimin Feng, Dehe Wang, Wei Deng, Fengdi Li, Zhiqi Song, Ziqiao Wang, Xiaosu Chen, Hua Qin, Qinyi Yu, Zhiqing Li, Shuxun Liu, Xun Xu, Nanshan Zhong, Xianwen Ren, Chuan Qin, Longqi Liu, Jian Wang, Xuetao Cao

**Affiliations:** 1 State Key Laboratory of Medicinal Chemical Biology, Institute of Immunology, College of Life Sciences, Nankai University, Tianjin, China; 2 Department of Immunology, Center for Immunotherapy, Peking Union Medical College, Chinese Academy of Medical Sciences, Beijing, China; 3 BGI-Shenzhen, Shenzhen, Guangdong China; 4 Institute of Laboratory Animal Sciences, Chinese Academy of Medical Sciences, Beijing, China; 5 Changping Laboratory, Beijing, China; 6 Institute of Immunology, Zhejiang University School of Medicine, Hangzhou, Zhejiang China; 7 National Key Laboratory of Immunity and Inflammation, Institute of Immunology, Navy Medical University, Shanghai, China; 8 Guangzhou Laboratory, Guangzhou, Guangdong China

**Keywords:** Innate immunity, Bioinformatics

## Abstract

How immune cells are spatiotemporally coordinated in the lung to effectively monitor, respond to, and resolve infection and inflammation in primed form needs to be fully illustrated. Here we apply immunocartography, a high-resolution technique that integrates spatial and single-cell RNA sequencing (scRNA-seq) through deconvolution and co-localization analyses, to the SARS-CoV-2-infected Syrian hamster model. We generate a comprehensive transcriptome map of the whole process of pulmonary infection from physiological condition, infection initiation, severe pneumonia to natural recovery at organ scale and single-cell resolution, with 142,965 cells and 45 lung lobes from 25 hamsters at 5 time points. Integrative analysis identifies that alveolar dendritic cell–T cell immunity hubs, where *Ccr7*^+^*Ido1*^+^ dendritic cells, *Cd160*^+^*Cd8*^+^ T cells, and *Tnfrsf4*^+^*Cd4*^+^ T cells physiologically co-localize, rapidly expand during SARS-CoV-2 infection, eliminate SARS-CoV-2 with the aid of *Slamf9*^+^ macrophages, and then restore to physiological levels after viral clearance. We verify the presence of these cell subpopulations in the immunity hubs in normal and SARS-CoV-2-infected hACE2 mouse models, as well as in publicly available human scRNA-seq datasets, demonstrating the potential broad relevance of our findings in lung immunity.

## Introduction

Immune surveillance in organs and tissues open to external stimuli is critical for rapid and effective responses in order to eliminate pathogens and protect the integrity of tissues. With the development of histology, multiple tissue structures and mechanisms that are relevant to immune surveillance have been revealed, including Peyer’s patches and Bronchus-Associated Lymphoid Tissues (BALT)^1–3^. A recent study revealed the importance of multicellular immunity hubs in human colorectal cancer by single-cell RNA sequencing (scRNA-seq)^4^. However, it remains unclear how immune surveillance is spatially implemented in lung due to the microscopic scale of the alveoli. As pulmonary infectious diseases, e.g., COVID-19 caused by severe acute respiratory syndrome coronavirus 2 (SARS-CoV-2) infection^5–7^, impose heavy burden to the global health, it is of urgent need to understand how pulmonary immune surveillance is implemented physiologically and how the host immune cells are dynamically reorganized to respond to infection, clear the etiological agents, and resolve inflammation. Multiple studies have provided important insights into the immunological and pathological mechanisms underlying SARS-CoV-2 infection based on human samples, experimental animal models and artificial intelligence^8–20^. However, such studies were limited by human sample types, such as autopsies, peripheral blood and bronchoalveolar lavage fluid, prohibiting the illustration of in situ immune cell coordination that is critical to viral clearance and inflammation resolution. Although experimental animals including human ACE2-transgenic mice (hACE2 mice) and hamsters have been used to model the pathogenesis of SARS-CoV-2 infection^8,9^, the spatial regulatory mechanisms underlying pulmonary immune surveillance, responding to and clearing SARS-CoV-2 infection, and resolving inflammation are still elusive because of the absence of high-resolution spatiotemporal information. Considering the importance of the dynamic reorganization and the intercellular communications in coordinating host immune responses to viral infection, we reason that the in situ information at the tissue level may reveal previously unknown cellular modules that orchestrate immune responses. And thus, the organ-scale high-resolution spatiotemporal analysis together with scRNA-seq provides a powerful tool for addressing this open question.

Although previous studies have unraveled dynamic cellular and transcriptomic changes in SARS-CoV-2-infected hamsters^21,22^, crucial mechanisms underlying SARS-CoV-2 clearance, inflammation resolution, and tissue repair are still elusive due to limited spatial resolution. Here we apply a high-resolution technique named immune cartography (immunocartography), which integrates our recently developed spatial transcriptomics technique Stereo-seq^23^ and scRNA-seq by single-cell deconvolution and co-localization analysis^24^, and use Syrian hamsters infected by SARS-CoV-2 as a model to simulate the whole process from physiological immune surveillance, infection, severe pneumonia, to viral clearance and inflammation resolution during two weeks. We applied immunocartography to 45 lung lobes of 25 hamsters and generated a comprehensive data atlas of the spatiotemporal landscape of in situ immune events proceeding within lung tissues during SARS-CoV-2 infection, clearance, and recovery. We found the presence of alveolar dendritic cell (DC)–T immunity hubs within normal hamster lungs, composed of *Ccr7*^+^*Ido1*^+^ DCs, *Cd160*^*+*^*Cd8*^*+*^ T cells, and *Tnfrsf4*^*+*^*Cd4*^*+*^ T cells, which may act as the pre-prepared niche in the alveoli defending against viral infection. When SARS-CoV-2 infection occurs, the immunity hubs expand in both size and number and recruit a new subpopulation of *Slamf9*^+^ macrophages (details about *Slamf9*^*+*^ macrophages can be found in our companion paper^25^). Adaptive T cell immunity is generally thought to start to work at ~one week after viral infection, but our findings suggest that T cells play critical roles in quick responses to viral infection as early as two days. Our findings, the immunocartography technology, and the comprehensive spatiotemporal lung immune landscape of viral infection may have profound implications in understanding antiviral immunity, inflammation and related disorders.

## Results

### Organ-scale immunocartography of hamster lungs before and after SARS-CoV-2 infection

**Fig. 1:**
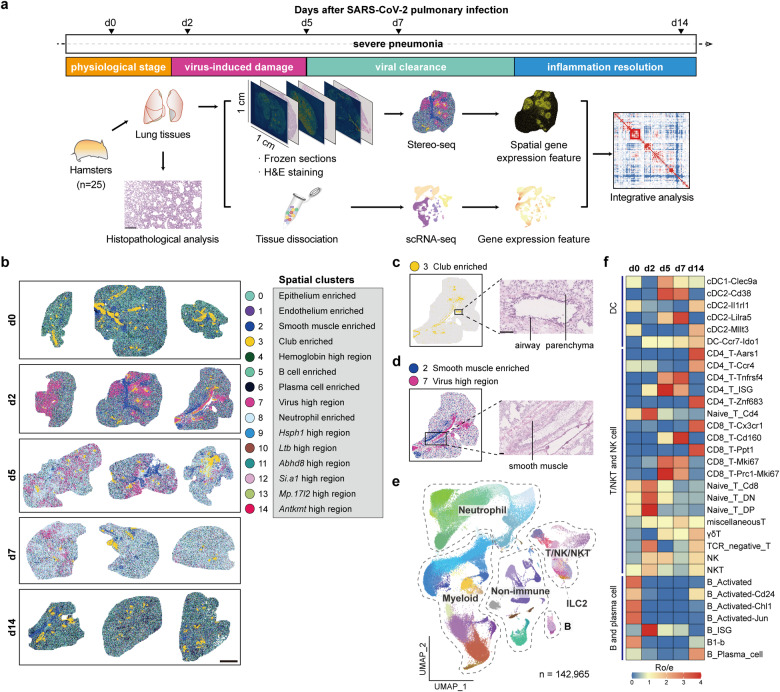
Single-cell spatiotemporal analysis of hamster lungs before and after SARS-CoV-2 infection. **a** Overview of the experimental design and analysis. **b** Unsupervised clustering of 15 spatial transcriptomic sections (three sections per timepoint) identifying 15 spatial clusters. Scale bar, 2 mm. **c**, **d** The spatial visualization of spatial cluster 3 representing club cell-enriched region (**c**), and spatial cluster 2 and 7 representing smooth muscle cell-enriched region and viral genes-highly expressed region (**d**) on a representative section, and the magnified Hematoxylin and Eosin (H&E) staining image of the framed region. Scale bar, 100 μm. **e** UMAP projection of hamster lung cells (*n* = 142,965) showing 79 cell subpopulations, including major cell types: non-immune (epithelium, endothelium, fibroblast and erythrocyte), myeloid, neutrophil, ILC2, T/NK/NKT and B cells. **f** Heatmap of the temporal distribution of DC, T/NK/NKT, B and plasma cell subpopulations at d0, d2, d5, d7 and d14. Ro/e > 1, enrichment; Ro/e < 1, depletion. The upper cutoff was set as 4.

To map the whole process of SARS-CoV-2 lung infection and clearance, we obtained a comprehensive cellular and molecular atlas by immunocartography, capturing the spatiotemporal changes of hamster lungs before and after authentic SARS-CoV-2 infection (Fig. 1a). Spatial transcriptomic data were generated from 45 lung slides covering 5 timepoints across two weeks during the infection (with each tissue area ~1 cm^2^, almost covering the entire lung lobe of the hamsters; at 500-nm resolution), and both the physiological and pathological changes were recorded (Supplementary Fig. S1a–d and Table S1). We also obtained scRNA-seq data for 142,965 single cells at the 5 timepoints (Fig. 1a). These data captured the spatiotemporal changes of hamster lungs before SARS-CoV-2 infection (d0), with acute tissue damages (d2), demonstrating acute immune responses (d5), exhibiting viral clearance (d7), and approaching inflammation resolution and tissue repair (d14).

We performed unsupervised spatial clustering and identified 15 clusters with distinct gene expression and histological patterns (Fig. 1b; Supplementary Fig. S1e and Table S2). Type II alveolar epithelial cells (ATII, *Sftpc*^+^) dispersed across lung tissues and were enriched in all spatial clusters (Supplementary Fig. S1e, f). Club cells (*Scgb3a2*^+^) were mainly detected in spatial cluster 3 and localized surrounding the airway as expected (Fig. 1c; Supplementary Fig. S1e). The *N* gene of SARS-CoV-2 was highly expressed in spatial cluster 7 with neutrophils (*S100a9*^+^) infiltrated, which accounted for the main proportion at d2 (Supplementary Fig. S1e–g). SARS-CoV-2 genes appeared to be dispersed across the entire lung lobe, and bronchial smooth muscle cells (*Myl9*^+^) were enriched in spatial cluster 2 (Fig. 1d; Supplementary Fig. S1e, f, h). Spatial clusters 5 and 6 were enriched by B cells (*Ighm*^+^) and plasma cells (*Jchain*^+^), respectively, which decreased after infection and partially recovered at d14 according to the quantitative analysis (Supplementary Fig. S1e, f). In contrast, neutrophils in spatial cluster 8, were mainly enriched at d5 and d7 (Supplementary Fig. S1e, f), which might account for severe pneumonia during the acute inflammation stage. In general, these data provide an elaborate map of the spatiotemporal changes of hamster lungs before and after SARS-CoV-2 infection.

The scRNA-seq data revealed 79 cell subpopulations according to unsupervised clustering and manual curation, covering non-immune cells (epithelium, endothelium, fibroblasts and erythrocytes), T/natural killer (NK)/natural killer T (NKT) cells, B cells, myeloid cells (alveolar macrophages, AMs; interstitial macrophages, IMs; monocytes/macrophages; dendritic cells, DCs; megakaryocytes), neutrophils, and type 2 innate lymphoid cells (ILC2) (Fig. 1e; Supplementary Fig. S2a and Table S3). These cell subpopulations together with the detection of SARS-CoV-2 RNAs demonstrated different dynamics during the infection process (Supplementary Fig. S2b, c and Table S4). Multiple cell types, including specific clusters of DCs, T cells and B cells, demonstrated a decreasing trend from d2 to d7, and then partially restored at d14, which were consistent with the observations of tissue damages and the following alleviation (Fig. 1f). There were also some cell types significantly expanding after SARS-CoV-2 infection (Fig. 1f).

### Alveolar DC–T immunity hubs revealed by immunocartography

To fully exploit the spatial information provided by Stereo-seq data and the cellular information provided by scRNA-seq data, we conducted an integrative analysis by employing Redeconve, an algorithm enabling spatial deconvolution analysis at fine granularity^24^. We observed consistency between epithelial and fibroblast subpopulations and their known histological localizations (Supplementary Fig. S2d). The deconvolution analysis also resulted in consistent observations as we stated above (Supplementary Fig. S2e), and thus proved the validity and enabled investigation of the spatiotemporal patterns of different immune and non-immune cell subsets before and after SARS-CoV-2 infection.

**Fig. 2:**
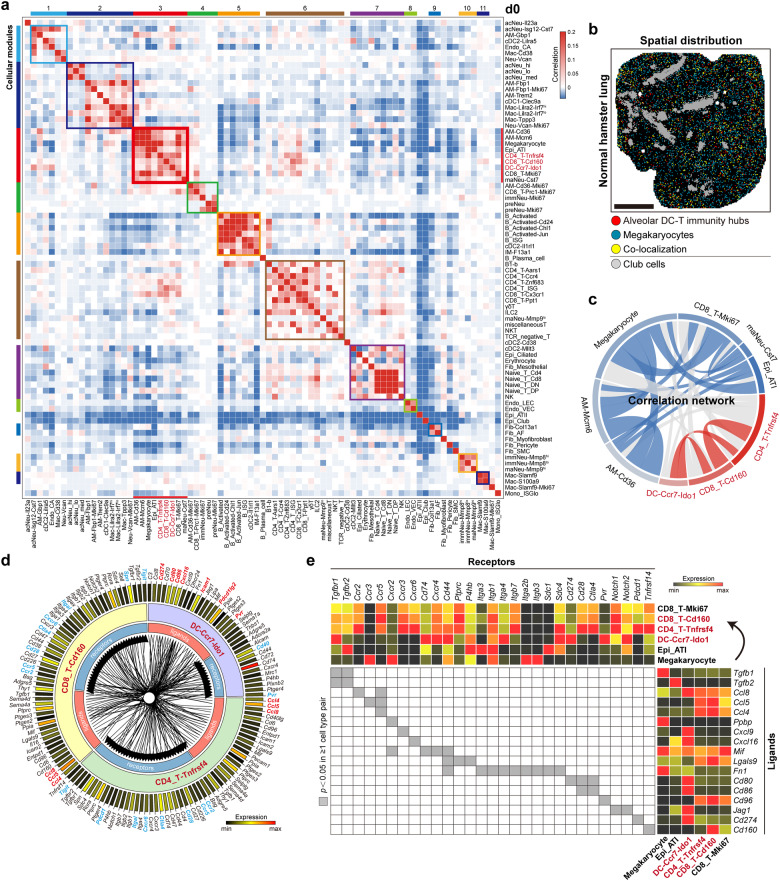
Identification of alveolar DC–T immunity hubs in hamster lungs. **a** Heatmap of the spatial correlation among 79 cell subpopulations at d0 revealing different modules. The module containing *Cd160*^+^*Cd8*^+^ T cells (module #3) is highlighted by the red frame. **b** The spatial distribution and the co-localization of alveolar DC–T immunity hubs (containing *Ccr7*^+^*Ido1*^+^ DCs, *Cd160*^+^*Cd8*^+^ T cells, and *Tnfrsf4*^+^*Cd4*^+^ T cells) and megakaryocytes in a slide of normal hamster lung (d0), and the spatial distribution of club cells was marked by grey spots. Scale bar, 2 mm. **c** Chord diagram showing the spatial correlation network among cell subpopulations in module #3 in **a** at d0. The thickness of the line indicates the correlation intensity. **d** The expression and putative signaling of selected receptors and their ligands among the key three cell types in the immunity hubs. The representative ligands are marked red, and the ligand-matched receptors are marked blue. **e** Right, heatmap of scRNA-seq expression of predicted ligands across cell types in the immunity hubs, *Mki67*^+^*Cd8*^+^ T cells, megakaryocytes, and ATI; top, heatmap of scRNA-seq expression of ligand-matched receptors expressed by these cell types; middle, heatmap of significant ligand–receptor pairs between ≥ 1 cell type pair.

The high spatial resolution, organ-scale field of view, and the transcriptome-wide features of our integrated Stereo-seq and scRNA-seq data enabled us to investigate the co-localization of different cell subpopulations before and after SARS-CoV-2 infection. Based on network clustering analysis, we identified 11 cellular modules. The cell subpopulations within the same module tended to be spatially co-localized and frequently appeared in physiological hamster lungs (Fig. 2a). Unexpectedly, modules #3 and #5 demonstrated features resembling lymphoid structures (Fig. 2a). Module #5 was composed of activated B cells and a group of *Ccr2*^+^*Sell*^+^ type 2 conventional dendritic cells (cDC2). Module #3 was mainly composed of *Ccr7*^+^*Ido1*^+^ migratory DCs, *Cd160*^+^*Cd8*^+^ T cells, proliferating *Cd8*^+^ T cells, *Tnfrsf4*^+^*Cd4*^+^ T cells, and *Cst7*^+^ mature neutrophils (Fig. 2a; Supplementary Fig. S3a). There were also AMs, type I alveolar epithelial cells (ATI) and megakaryocytes in module #3, suggesting the alveolar localization of the cells within this module (Fig. 2a). Because of the significant increase of cell number after SARS-CoV-2 infection (Supplementary Fig. S3b), as well as the robust co-localization of *Ccr7*^+^*Ido1*^+^ DCs, *Cd160*^+^*Cd8*^+^ T cells, and *Tnfrsf4*^+^*Cd4*^+^ T cells through the whole process of SARS-CoV-2 infection (see below), we then sought to investigate the features of such stable DC–T immunity hubs. These DC–T immunity hubs showed co-localization with alveolar epithelial cells and megakaryocytes instead of club cells in the airway (Fig. 2b, c). Based on CellChat analysis to infer intercellular communications of the co-localized cell subpopulations^26^, we studied the potential interactions among the cell types in the DC–T immunity hubs and alveoli-located ATI and megakaryocytes (Supplementary Fig. S3c). *Cd160*^+^*Cd8*^+^ T cells and *Tnfrsf4*^+^*Cd4*^+^ T cells together with *Ccr7*^+^*Ido1*^+^ DCs displayed potential intercellular interactions through chemokines, co-stimulatory factors and adhesion molecules (Fig. 2d). Interactions may also exist among these three cell types, megakaryocytes and ATI through galectin, FN1, TGF-β, MIF, CXCL, CD96, and CD80/CD86 pathways (Fig. 2e; Supplementary Fig. S3d, e). *Ccr7*^+^*Ido1*^+^ DCs and ATI might recruit and interact with *Cd160*^+^*Cd8*^+^ T cells and *Tnfrsf4*^+^*Cd4*^+^ T cells through *Cxcl16/Cxcr6* and *Jag1/Notch1* (Fig. 2e).

**Fig. 3:**
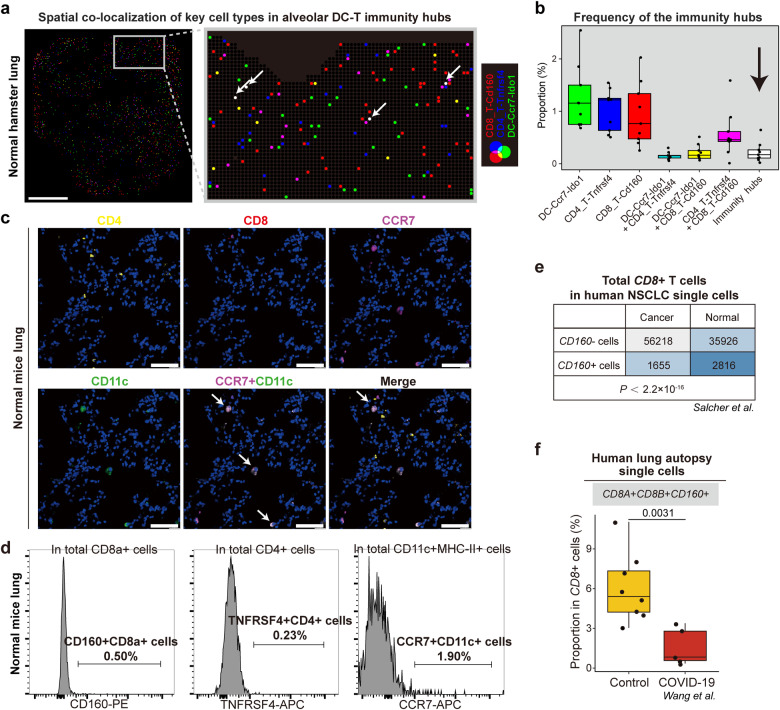
The spatial visualization and validation of the alveolar DC–T immunity hubs under physiological conditions. **a** Spatial distribution showing *Ccr7*^+^*Ido1*^+^ DCs, *Cd160*^+^*Cd8*^+^ T cells, and *Tnfrsf4*^+^*Cd4*^+^ T cells in a slide of normal hamster lung (d0). Co-localization is demonstrated by blending three RGB colors. A selected view is magnified to show the white dots that represented the immunity hubs. Scale bar, 2 mm. **b** Boxplot showing the quantitative proportion of *Ccr7*^+^*Ido1*^+^ DCs, *Cd160*^+^*Cd8*^+^ T cells, *Tnfrsf4*^+^*Cd4*^+^ T cells, and their co-localization in nine slides of normal hamster lung (d0). Boxes were colored corresponding to panel **a**. Data are represented as mean ± SEM. Center line, median; box bounds, first and third quartiles; whiskers, 1.5 times the interquartile range. **c** Representative images of normal hACE2 mice lungs showing CD4, CD8, CCR7 and CD11c protein detected by immunofluorescence. Co-localizations are indicated by arrows. Scale bars, 50 μm. **d** Histogram plots showing the detection of CCR7^+^CD11c^+^ cells, CD160^+^CD8^+^ T cells, and TNFRSF4^+^CD4^+^ T cells in the normal lung of C57BL/6J mice. Values represent the mean percentage (*n* = 5). **e** 2 × 2 contingency table displaying the number of *CD160-negative and CD160-positive cells* in total *CD8*^+^ T cells in scRNA-seq data of human NSCLC samples of normal and cancer tissues. Chi-square test. **f** Boxplot showing the proportion of *CD8A*^*+*^*CD8B*^*+*^*CD160*^*+*^ cells in all *CD8*^*+*^ cells in scRNA-seq data of human lung autopsy samples of control (*n* = 8) and COVID-19 patients (*n* = 5). Control: pulmonary bulla or lung cancer, and these samples were pathologically confirmed to be inflammation-free. Data are represented as mean ± SEM. Two-sided Wilcoxon test.

The proportion of these immunity hubs was less than 1% in lung tissues under physiological conditions, estimated by the proportion of the spots containing *Ccr7*^+^*Ido1*^+^ DCs, *Cd160*^+^*Cd8*^+^ T cells, and *Tnfrsf4*^+^*Cd4*^+^ T cells (Fig. 3a, b; Supplementary Table S5). Such immunity hubs may be present in normal mice as illustrated by immunofluorescent staining (Fig. 3c). The presence of CCR7^+^ DCs, CD160^+^CD8^+^ T cells, and TNFRSF4^+^CD4^+^ T and in the lungs of normal mice was also verified by flow cytometry (Fig. 3d; Supplementary Fig. S3f). According to a human scRNA-seq dataset of non-small cell lung cancer (NSCLC)^27^, we found that the proportion of *CD160*^*+*^*CD8*^*+*^ T cells in total *CD8*^*+*^ T cells in the control samples was significantly higher compared to that in NSCLC samples (Fig. 3e), potentially linking the inadequacy of pulmonary *CD160*^*+*^*CD8*^*+*^ T cells to lung tumorigenicity. We also validated the presence of *CD160*^*+*^*CD8*^*+*^ T cells based on a publicly available human autopsy scRNA-seq dataset^28^, and the lung autopsy samples from COVID-19 patients demonstrated fewer *CD160*^*+*^*CD8*^*+*^ cells than controls (Fig. 3f).

### Responses of the alveolar DC–T immunity hubs to SARS-CoV-2 infection

**Fig. 4:**
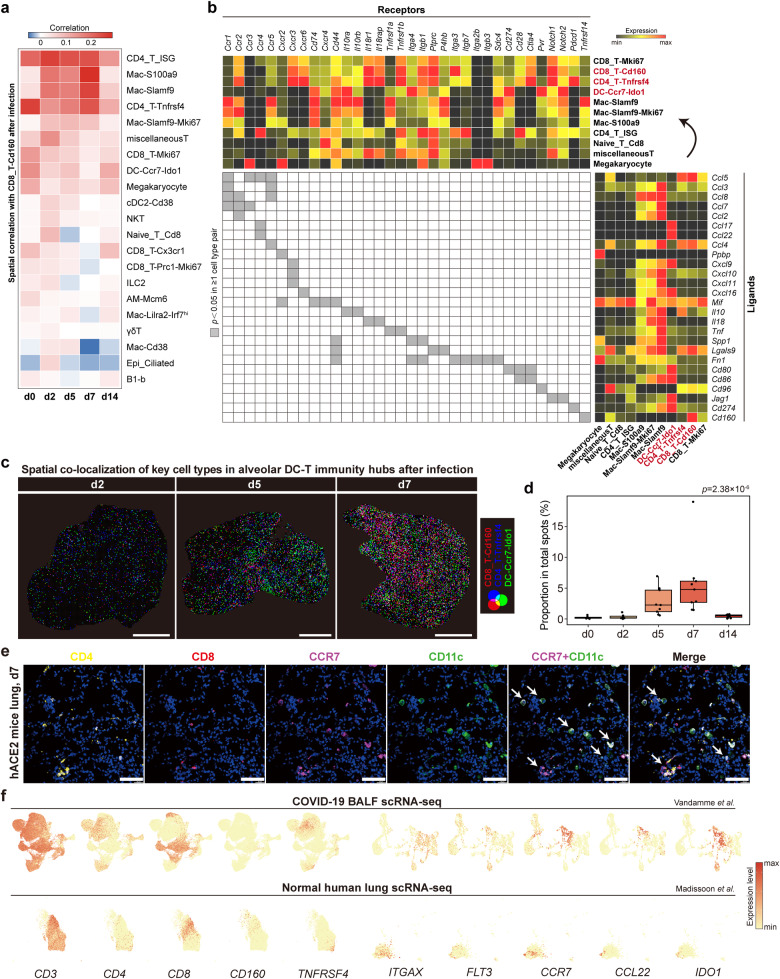
Responses of the alveolar DC–T immunity hubs to SARS-CoV-2 infection. **a** Heatmap of spatial correlation among multiple cell subpopulations with *Cd160*^+^*Cd8*^+^ T cells at each timepoint. Cell subpopulations were the union of the top 15 subpopulations with the highest correlation with *Cd160*^+^*Cd8*^+^ T cells at d2, d5 and d7. **b** Right, heatmap of scRNA-seq expression of predicted ligands across specific cell subpopulations with high correlation with *Cd160*^+^*Cd8*^+^ T cells (see Materials and methods); top, heatmap of scRNA-seq expression of ligand-matched receptors expressed by these cell types; middle, heatmap of significant ligand–receptor pairs between ≥ 1 cell type pair. **c** Spatial distribution showing *Ccr7*^+^*Ido1*^+^ DCs, *Cd160*^+^*Cd8*^+^ T cells, and *Tnfrsf4*^+^*Cd4*^+^ T cells after SARS-CoV-2 infection, represented by a slide at d2, d5 and d7, respectively. Co-localization is demonstrated by blending three RGB colors. Scale bars, 2 mm. **d** Box plot showing the proportion of the immunity hubs in total Stereo-seq bin80-spots of nine lung slides at each timepoint. Data are represented as mean ± SEM (*n* = 9 slides per timepoint). Center line, median; box bounds, first and third quartiles; whiskers, 1.5 times the interquartile range. Kruskal–Wallis test. **e** Representative images of hACE2 mice lungs showing CD4, CD8, CCR7 and CD11c protein at d7 after SARS-CoV-2 infection detected by immunofluorescence. Co-localizations are indicated by arrows. Scale bars, 50 μm. **f** UMAP projection showing the expression of the signature genes of *CD160*^+^*CD8*^+^ T cells, *TNFRSF4*^+^*CD4*^+^ T cells and *CCR7*^+^*CCL22*/*IDO1*^+^ DCs in public scRNA-seq database of normal human lung tissues (downloaded from https://covid19.cog.sanger.ac.uk/madissoon19_lung.processed.h5ad) and BALF of COVID-19 patients (downloaded from https://covid19.cog.sanger.ac.uk/submissions/release1/BALF_VIB-UGent_processed_cleaned.h5ad).

During SARS-CoV-2 infection, the core component of the DC–T immunity hubs, *Cd160*^+^*Cd8*^+^ T cells, maintained stable spatial co-localization with *Tnfrsf4*^+^, proliferating CD8^+^ T cells, *Ccr7*^+^*Ido1*^+^ migratory DCs and megakaryocytes, and enhanced spatial co-localization with *Isg*^+^CD4^+^ T cells, *S100a9*^+^ macrophages, *Slamf9*^+^ macrophages, *Cd38*^+^ macrophages, and *Cd38*^+^ cDC2 (Fig. 4a), suggesting an expansion state of the immunity hubs when responding to SARS-CoV-2 infection. The spatial correlation network indicated potential relationship between *Cd160*^+^*Cd8*^+^ T cells with diverse inflammatory myeloid cells at d2, d5 and d7 (Fig. 4a). Ligand–receptor analysis supported the participation of multiple cell types including *Slamf9*^+^ macrophages and *S100a9*^+^ macrophages in the expansion of such immunity hubs responding to SARS-CoV-2 infection (Fig. 4b; Supplementary Fig. S4a–c). The dynamic increase of the immunity hubs could also be intuitively observed in Stereo-seq slides after infection (Fig. 4c, d). *Ccr7*^+^*Ido1*^+^ DCs, *Cd160*^+^*Cd8*^+^ T cells, and *Tnfrsf4*^+^*Cd4*^+^ T cells all increased after infection, and were viral RNA-positive (Supplementary Fig. S4d), suggesting direct involvement of these immunity hubs against SARS-CoV-2 infection. We then verified the presence of spatial co-localization of CD4, CD8, CD11c, and CCR7 in hACE2 mice after SARS-CoV-2 infection by immunofluorescent staining (Fig. 4e). Meanwhile, we verified the existence of *CCR7*^*+*^*CCL22/IDO1*^*+*^ DCs, *CD160*^*+*^*CD8*^*+*^ T cells, and *TNFRSF4*^*+*^*CD4*^*+*^ T cells in public scRNA-seq datasets of normal human lung tissues^29^ and bronchoalveolar lavage fluid (BALF) of COVID-19 patients in COVID-19 Cell Atlas (Fig. 4f), suggesting the potential role of these cell types in lung immune surveillance and response to SARS-CoV-2 pulmonary infection.

### Rapid expansion of *Cd160*^+^*Cd8*^+^ T cells in lung to clear SARS-CoV-2 infection

*Cd160*^+^*Cd8*^+^ T cells accumulated at d2 and d5, and peaked at d7, different from the temporal tendency of total T cells (Supplementary Fig. S3b). The *Cd160*^+^*Cd8*^+^ T cells belonged to αβT cells, highly expressed granzyme genes and demonstrated an exhausted phenotype (Supplementary Fig. S3a). These cells expressed the low level of *Sell* and high levels of *Cd69* and *Itgae* (Supplementary Fig. S3a). Similar to these *Cd160*^+^*Cd8*^+^ T cells, the T cell cluster CD8_T-Ppt1 also expressed high levels of *Cd69* and *Itgae*, but these cells were *Cd160*-negative (Supplementary Fig. S3a). *Cd160*^+^*Cd8*^+^ T cells spatially correlated with multiple cell types under physiological conditions, including *Ccr7*^+^*Ido1*^+^ DCs, *Tnfrsf4*^+^*Cd4*^+^ T cells, ATI, megakaryocytes and also a subset of *Cd36*^+^ AMs (the most frequent subpopulation of AMs at d0; Supplementary Fig. S5a), suggesting their alveolar positioning. Upon SARS-CoV-2 infection, *Slamf9*^+^ macrophages, *S100a9*^+^ macrophages, *Cd38*^+^ macrophages, *Cd38*^+^ cDC2, and ciliated cells showed higher correlation with *Cd160*^+^*Cd8*^+^ T cells, while club cells, lymphatic endothelial cells (LECs) and vein endothelial cells (VECs) did not show higher positive correlation with *Cd160*^+^*Cd8*^+^ T cells after infection (Supplementary Fig. S5a).

**Fig. 5:**
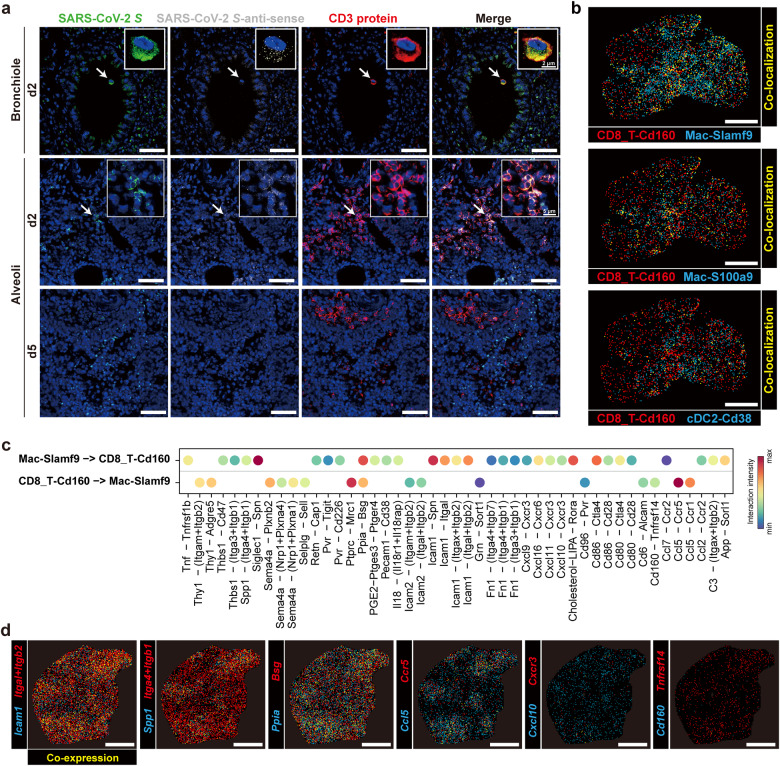
The orchestra of *Cd160*^+^*Cd8*^+^ T cells and *Slamf9*^+^ macrophages after SARS-CoV-2 infection. **a** Representative images of hACE2 mice lungs showing SARS-CoV-2 positive-sense RNA (SARS-CoV-2 *S*), negative-sense RNA (SARS-CoV-2 *S*-anti-sense) and CD3 protein at d2 and d5 detected by immunofluorescence and RNAscope. Top, bronchiole regions; bottom, alveoli regions. Co-localizations are indicated by arrows and magnified images. Scale bars, 50 μm. **b** Spatial distribution showing the co-localization of *Cd160*^+^*Cd8*^+^ T cells and representative cell types at a slide at d5. Top, *Slamf9*^+^ macrophages; middle, *S100a9*^+^ macrophages; bottom, *Cd38*^+^ cDC2. Scale bars, 2 mm. **c** Bubble chart showing the interaction intensity of putative ligand–receptor pairs between *Cd160*^*+*^*Cd8*^*+*^ T cells and *Slamf9*^*+*^ macrophages. The left side of the arrows represents the sender cell type, and the right side represents the receiver cell type. **d** Spatial expression and co-localization of the selected paired ligand and receptor genes in **c** represented by a lung slide at d7. Scale bars, 2 mm.

Consistent with the potential anti-viral role of *Cd160*^+^*Cd8*^+^ T cells, viral RNAs were detected in a small proportion of these *Cd160*^+^*Cd8*^+^ T cells at d5 and d7 according to our scRNA-seq data (Supplementary Fig. S4d), which was consistent with that observed in the spatial data (Supplementary Fig. S5b). The co-localization of *Cd160*^+^*Cd8*^+^ T cells with viral RNAs at d2, d5 and d7 was also shown by Stereo-seq data (Supplementary Fig. S5c). We confirmed that not only SARS-CoV-2 genomic RNAs existed in T cells but also the viral negative-sense RNAs were co-localized around bronchiole and alveoli regions at d2 based on RNAscope and immunofluorescence of hACE2 mice lung slides after SARS-CoV-2 infection (Fig. 5a). Cell death signal *Casp3* was observed in *Cd160*^+^*Cd8*^+^ T cells according to the Stereo-seq data (Supplementary Fig. S5d). We further verified the co-detection of viral S protein, T cell markers and cleaved caspase-3 by immunofluorescent staining (Supplementary Fig. S5e). Besides, the enrichment of *CASP3*^+^*CD8*^+^*CD160*^+^ cells was observed in the lung autopsy samples of COVID-19 patients (Supplementary Fig. S5f). These data imply the potential invasiveness of SARS-CoV-2 to T cells, and suggest that *Cd160*^+^*Cd8*^+^ T cells could resist the invasion by virus and then proliferate, thus indicating the anti-SARS-CoV-2 role of these *Cd160*^+^*Cd8*^+^ T cells.

The aggregation of multiple immune cell types with *Cd160*^+^*Cd8*^+^ T cells during infection can be observed in Stereo-seq slides (Fig. 5b). To analyze their potential intercellular interactions, we performed ligand–receptor analysis which showed that these *Cd160*^+^*Cd8*^+^ T cells highly expressed *Ccl5*, while *Slamf9*^+^ macrophages highly expressed *Ccr5* (Fig. 5c). *Slamf9*^+^ macrophages highly expressed *Cxcl16*, *Cxcl11 and Cxcl10*, while *Cd160*^+^*Cd8*^+^ T cells highly expressed the corresponding receptor gene *Cxcr6* and *Cxcr3* (Fig. 5c). Spatially, such specific ligand–receptor pairs were co-expressed according to our Stereo-seq data (Fig. 5d), indicating the chemotaxis and the adhesion of these cells after viral infection. We also observed the co-expression and adjacent distribution of *Cd160/Tnfrsf14* at d7 (Fig. 5d), indicating the regulation of *Cd160*^+^*Cd8*^+^ T cell function by these *Slamf9*^+^ macrophages.

### Restoration of DC–T immunity hubs at the late stage of infection

Both scRNA-seq and Stereo-seq data revealed that SARS-CoV-2 RNAs were nearly absent from hamster lungs at d14, and the alveolar DC–T immunity hubs containing *Ccr7*^+^*Ido1*^+^ DCs, *Cd160*^+^*Cd8*^+^ T cells, and *Tnfrsf4*^+^*Cd4*^+^ T cells restored to physiological levels at d14 (Supplementary Fig. S6a–c). Similarly, immune infiltration and lung injury were also attenuated at d14 as revealed by pathological observations (Supplementary Fig. S1d). Accompanying with the diminishment of SARS-CoV-2, multiple myeloid cells including *S100a9*^+^ macrophages and *Slamf9*^+^ macrophages also diminished from these immunity hubs (Supplementary Fig. S6a). The number of *Cd160*^+^*Cd8*^+^ T cells and *Slamf9*^+^ macrophages rapidly increased after SARS-CoV-2 infection but returned to physiological levels after viral clearance, suggesting their potential roles in combating SARS-CoV-2 infection. These data indicate that the *Cd160*^+^*Cd8*^+^ T cells-based DC–T immunity hubs may serve as a pre-prepared defensive niche to rapidly sense and respond to SARS-CoV-2 infection, and significantly expand in size and number, working in conjunction with multiple other immune cell types to clear the SARS-CoV-2 infection.

## Discussion

In lung, lymphoid aggregations are often found in close proximity to bronchi, i.e., inducible BALT (iBALT). iBALT are characterized by the aggregation of GL7^+^ germinal center B cells, T cells and DCs, and develop at the late stage of chronic immune stimulation, e.g., 17 days post influenza virus infection^30,31^. Different from iBALT, our high-resolution spatial data suggested the presence of alveoli-located DC–T immunity hubs, with *Cd160*^+^*Cd8*^+^ T cells spatially co-localized with CD4^+^ T cell subsets and *Ccr7*^+^*Ido1*^+^ migratory DCs in normal hamster lungs and responsive to lung acute infection. Our previous study identified a cluster of *CCR7*^+^*LAMP3*^*+*^*IDO1*^*+*^*CD274*^*+*^ activated DCs in hepatocellular carcinoma^32^, which possess the potential to migrate from tumors to lymph nodes. The transcriptome signatures of these *CCR7*^+^*LAMP3*^*+*^ *IDO1*^*+*^*CD274*^*+*^ activated DCs are reminiscent of mature DCs enriched in immunoregulatory molecules (mregDCs)^33^ and play important roles in responses to PD-1 blockade of hepatocellular carcinomas^34^. Recently, Chen et al. applied spatial profiling techniques to lung cancer samples and uncovered mregDC-residing stem–immunity hubs associated with beneficial responses to immunotherapy^35^. *Ccr7*^+^*Ido1*^+^ DCs within the immunity hubs identified in our current study not only showed expression similarity with *CCR7*^+^*LAMP3*^*+*^ *IDO1*^*+*^*CD274*^*+*^ activated DCs or mregDCs identified in human cancers, but also showed similar spatial co-localization with CD4^+^ and CD8^+^ T cells. These immunity hubs in the lung may serve as sentinel to ensure rapid immune responses defensive to viral infection. Because of technical limitation, such immunity hubs were hard to be detected by previous traditional technologies. Therefore, our findings of such DC–T immunity hubs based on immunocartography may have broad roles in a wide range of mammals (from humans to hamsters and mice) and in various settings including pulmonary physiological conditions, viral infections, and cancers. Further investigations are needed to substantiate the function of the immunity hubs under both physiological and pathological conditions.

Adaptive T cell immune response is traditionally accepted to play critical roles almost one week later after a novel viral pathogen infects. Our Stereo-seq and scRNA-seq data captured the spatiotemporal pulmonary changes and identified that a previously undescribed group of *Cd160*^+^*Cd8*^+^ T cells may play important roles in rapidly responding to and clearing SARS-CoV-2 infection, suggesting the function of T cells at the early stage of infection and the importance of in situ spatial profiling. The dynamics and mechanisms of the alveolar DC–T immunity hubs in responding and defensive to SARS-CoV-2 at the early stage of viral infection and resolving inflammation at the late stage of viral infection may be summarized as follows: (1) *Ccr7*^+^*Ido1*^+^ DCs, *Cd160*^+^*Cd8*^+^ T cells, and *Tnfrsf4*^*+*^*Cd4*^*+*^ T cells constitute alveolar immunity hubs in hamster lungs at a low level under physiological conditions for immune surveillance; (2) stimulated by SARS-CoV-2 infection, the immunity hubs expand and increase by assembling diverse types of other immune cells, especially *S100a9*^+^ and *Slamf9*^+^ macrophages; (3) *Cd160*^+^*Cd8*^+^ T cells and *Slamf9*^+^ macrophages proliferate actively within the expanded immunity hubs to win the combat against SARS-CoV-2 intra-host transmission; (4) *Cd160*^+^*Cd8*^+^ T cells and *Slamf9*^+^ macrophages interact with each other and probably cooperate to clear SARS-CoV-2. The conditions sufficient or necessary for the induction of these immune cells and their spatiotemporal reallocation are still unknown, and further studies are needed to illustrate the metabolic, epigenomic, physical factors necessary for their induction and function.

Our findings are data-driven and depend on bioinformatic analyses. Although we have combined different experimental methods to validate our findings in samples from mice and clinical samples, the limited availability of antibodies targeted to different proteins in hamster, mouse, and human samples prevents precise validation of findings derived from the high-resolution single-cell spatiotemporal data. While our study is limited by the observational nature and future studies are needed to confirm and substantiate these observations, it is worth noting that such findings were directly derived from in vivo SARS-CoV-2 infection through the lens of single-cell immunocartography. When a new infectious disease emerged, it is of urgent need to quickly identify those host factors that are important to limit, clear the pathogens and resolve their damages. However, because of the complexity of in vivo infection and the related host immune responses, traditional technologies cannot address such challenges due to the limitations of spatial, temporal, and cellular resolution, limiting the development of anti-infectious measurements and therapies. Although scRNA-seq provides sufficient resolution at the single-cell level, the loss of spatiotemporal information and the selection biases towards live cells (survivor biases) introduced during scRNA-seq library preparation as we illustrated in this study prohibit its power to directly identify critical host factors important for pathogen clearance and inflammation resolution. Our study demonstrated the power of single-cell immunocartography in illustrating the molecular and cellular details of in vivo biological events. Therefore, single-cell immunocartography will be a powerful technique to boost studies including inflammatory damages, infectious diseases, tumors, and any other questions related to cell death.

In summary, our study applied a technique of immunocartography, and depicted the spatial allocation of diverse cell types of hamster lungs and their spatiotemporal dynamics after SARS-CoV-2 infection. We identified the alveolar DC–T immunity hubs under physiological conditions, within which *Cd160*^+^*Cd8*^+^ T cells respond as early as two days after SARS-CoV-2 infection, and clear virus via orchestration with *Slamf9*^+^ macrophages. These findings provide actionable hypotheses for precisely understanding the mechanisms underlying pulmonary immune surveillance and viral clearance. The rich information generated by the immunocartography analysis of Stereo-seq and scRNA-seq data provides a comprehensive and insightful resource for understanding infectious diseases and developing anti-viral therapies in the future.

## Materials and methods

### Ethics statement

At the Institute of Laboratory Animal Science (ILAS) of Chinese Academy of Medical Sciences, the animal biosafety level 3 (ABSL-3) facility was used to accomplish all the experiments with Syrian hamsters (male and female, aged 8–10 weeks) and hACE2 mice (male and female, aged 6–11 months). All experiments were implemented according to the Animal Welfare Act and other regulations associated with animals and experiments. The Institutional Animal Care and Use Committee (IACUC) of the ILAS, Peking Union Medical College & Chinese Academy of Medical Sciences, evaluated and gave permission to all the protocols in these studies, including animal experiments (Approval number QC21003).

### Viruses

The SARS-CoV-2 virus (accession number is MT093631.2, SARS CoV-2/WH-09/human/2020/CHN) was put into use in this study and propagated in Vero E6 cells and incubated in Dulbecco’s modified Eagle’s medium (Invitrogen, Carlsbad, United States) supplemented with 10% fetal bovine serum (Gibco, Grand Island, United States) at 37 °C with 5% carbon dioxide.

### Experimental hamsters

Specific-pathogen-free, 8–10-week-old male and female Syrian hamsters were intranasally inoculated with SARS-CoV-2 stock virus at 10^5^ 50% tissue culture infectious dose (TCID_50_) per mL (0.1 mL per animal)^36^. Hamsters intranasally inoculated with an equal volume of PBS were used as the healthy control (Ctrl) group. Hamsters were continuously monitored to record clinical symptoms, body weight, responses to external stimuli and death. No animals were needed to be sacrificed to avoid undue suffering. Lung tissues of the infected group were collected at 2, 5, 7 and 14 days after infection (*n* = 5 per timepoint), and those of the Ctrl group were collected at 2 days (*n* = 5) after PBS administration. In the five lung lobes of each hamster, one was subjected to histopathological analysis, one was subjected to the detection of viral load, and the other three were used for scRNA-seq or Stereo-seq.

### Experimental mice

Specific-pathogen-free, 6–8-week-old female C57BL/6J mice were obtained from Beijing Vital River Laboratory Animal Technology Co., Ltd (Beijiing, China). Specific-pathogen-free, 6–11-month-old male and female hACE2 mice were obtained from the Institute of Laboratory Animal Sciences, Chinese Academy of Medical Sciences as described in our previous study^8^. The hACE2 mice were intranasally inoculated with SARS-CoV-2 stock virus at a dosage of 10^5^ TCID_50_, and those intranasally inoculated with an equal volume of PBS were used as healthy control (d0). The infected hACE2 mice were continuously observed to record body weight, clinical symptoms, responses to external stimuli and death. Mice were dissected after certain days to collect lung tissues for further experiments.

### H&E staining

Hamster lungs were fixed in 10% formalin buffer saline (HT501128, Sigma-Aldrich) for 24 h at room temperature before paraffin embedding, and paraffin sections (4 µm in thickness) were applied following the routine operating procedure. The adjacent frozen sections (8–10 µm in thickness) of those sections used for Stereo-seq were fixed in acetone. All the tissue sections were stained with H&E. The pathological findings were carefully observed using an Olympus microscope. The pathological scorings of all hamster lungs are listed in Supplementary Table S1.

### In situ RNA hybridization

In situ RNA hybridization was performed using the Advanced Cell Diagnostics RNAscope Multiplex Fluorescent Detection kit v2 (323100, Bio-Techne) according to the manufacturer’s instructions. Staining of hamster lung specimens was performed using paraffin-embedded sections (3–4 µm in thickness). For multiplex staining, the following probes were used: V-nCoV2019-S 848561-C2, V-nCoV2019-S 848561-C1 and V-nCoV2019-S-sense 845701-C2. V-nCoV2019-S 848561-C1 and V-nCoV2019-S-sense 845701-C2 were co-detected with Anti-CD3 antibody [SP7] (ab16669, Abcam). Slides were counterstained with Mounting Medium With DAPI-Aqueous (ab104139, Abcam). Mounted slides were imaged on a Leica DMi8 fluorescent microscope (Leica Biosystems).

### Multiplex immunofluorescence staining

All collected lung tissues from hACE2 mice were fixed in 10% formalin buffer solution (HT501128, Sigma-Aldrich), and paraffin-embedded sections (3–4 µm in thickness) were prepared according to routine operating procedure^37,38^. The immunofluorescence staining was performed using a PANO IHC kit (10004100100, Panovue, Beijing, China) following the manufacturer’s instructions^39^. Different primary antibodies were sequentially applied to examine specific cell markers, including SARS-CoV-2 (COVID-19) Spike S1 antibody [HL6] (GTX635654, 1:100; GeneTex), Anti-CD4 antibody [EPR19514] (ab183685, 1:800; Abcam), CD8α (D4W2Z) XP® Rabbit mAb (98941, 1:200; Cell Signaling Technology) and Cleaved Caspase-3 (Asp175) Antibody (9661, 1:400; Cell Signaling Technology); CD8α (D4W2Z) XP® Rabbit mAb (98941, 1:400; Cell Signaling Technology), Anti-CD4 antibody [EPR19514] (ab183685, 1:2000; Abcam), CD11c (D1V9Y) Rabbit mAb (97585s, 1:300; CST), and Anti-CCR7 antibody [EPR23192-57] (ab253187, 1:500; Abcam), followed by HRP-conjugated secondary antibody incubation and tyramide signal amplification (TSA). The slides were microwave-treated after each cycle of TSA. Nuclei were stained with 4′-6′-diamidino-2-phenylindole (DAPI; Sigma-Aldrich) after antigen labeling. Stained slides were scanned using the 3D-histech (PANNORAMIC, 3DHISTECH, Hungary), which captures fluorescent spectra with identical exposure times using DAPI, FITC, TRICT and Cy5 channels, and the scans were combined to build a single stacked image.

### Flow cytometry

Lung tissues were first rinsed with PBS, minced into small pieces by mechanical dissociation, and incubated at 37 °C for 30 min in the digestion buffer, containing 2 mg/mL Collagenase I (17018029, Gibco), 2 mg/mL Collagenase IV (17104019, Gibco), 0.2 mg/mL DNase I (DN25, Sigma-Aldrich) and 10% FBS in RPMI 1640 medium. After this, the tissue digestion was blocked by adding RPMI 1640 medium containing 20% FBS and 1 mM EDTA, followed by filtration through a 40-µm cell strainer and centrifugation for 5 min at 500× *g* at 4 °C. Red blood cells were lysed in Red blood cell lysis buffer (R1010, Solabio) for 5 min, and washed with PBS buffer, followed by filtration through a 40-µm cell strainer and centrifugation for 5 min at 500× *g* at 4 °C. 5 × 10^5^ cells were isolated for each sample, which were Fc-blocked with CD16/32 antibody (101302, Biolegend). For T cell experiments, cells were stained with Zombie UV viability dye (423107, Biolegend) for 10 min at room temperature, and then were stained with surface marker CD45-APC/Fire 750, CD3-BV421, CD4-FITC, CD8a-BV605, CD160-PE and TNFRSF4(OX40)-APC for 30 min at 4 °C. For DC experiments, cells were stained with Zombie UV viability dye (423107, Biolegend) for 10 min at room temperature, and then were stained with surface marker CD45-APC/Fire 750, CD64-PE/Cy7, MerTK-FITC, CD11c-BV421, MHC-II-PE and CCR7-APC for 30 min at 4 °C. Then, the stained cells were washed twice for acquisition on a BD LSR Fortessa. Data were analyzed by Flowjo.

### scRNA-seq library preparation and sequencing

#### Single-cell suspension preparation

In the ABSL-3 laboratory, fresh lung tissues were first rinsed with PBS, minced into small pieces by mechanical dissociation, and incubated at 37 °C for 30 min in 10 mL of digestion buffer, containing 2 mg/mL Collagenase I (17018029, Gibco), 2 mg/mL Collagenase IV (17104019, Gibco), 0.2 mg/mL DNase I (DN25, Sigma-Aldrich) and 10% FBS in RPMI 1640 medium. After this, the tissue digestion was blocked by adding 3 mL of RPMI 1640 medium containing 20% FBS and 1 mM EDTA, followed by filtration through a 40-µm cell strainer and centrifugation for 5 min at 500× *g* at 4 °C. Red blood cells were lysed in Red blood cell lysis buffer (R1010, Solabio) for 5 min, and washed with PBS buffer, followed by filtration through a 40-µm cell strainer and centrifugation for 5 min at 500× *g* at 4 °C. Pellets were resuspended in cell resuspension buffer at a concentration of 1000 cells/μL for library preparation.

#### Library construction and sequencing

DNBelab C Series Single-Cell Library Prep Set (1000021082, MGI) was used as described previously^40^. In brief, high-quality single-cell suspension was used for droplet generation, cell lysis and mRNA capture by microbeads were performed in the droplets, then emulsion breakage, beads collection, reverse transcription, finally the cDNA and droplet index were amplification to generate cDNA library and droplet index library respectively. All the processes were performed in the ABSL-3 laboratory till the cDNA products were gained. Library concentration was measured by use of Qubit™ ssDNA Assay Kit (Q10212, Thermo Fisher Scientific) and sequenced by DNBSEQ T10 sequencer in the China National GeneBank (Shenzhen, China).

### scRNA-seq data processing

#### Raw data processing

For DNBelab C4 data, PISA (v0.7, https://github.com/shiquan/PISA) was used for sequencing reads filtering and demultiplexing. The reference genome FASTA file and gene annotation file (GTF) for the Mesocricetus auratus genome (BCM_Maur_2.0, NCBI RefSeq assembly: GCF_017639785.1) and the SARS-CoV-2 genome (NCBI Reference Sequence: NC_045512.2) were downloaded from NCBI. These files were then integrated together as an inhouse hamster reference genome. The alignment tool STAR (v2.7.9a)^41^ was applied to map reads with the reference genome.

#### scRNA-seq data

The number of detected genes, the total UMI counts and proportion of mitochondrial gene counts per cell were used for quality control. Low quality cells with < 1000 UMI counts, < 300 detected genes, or > 5% mitochondrial gene counts were filtered. Cells with > 60,000 UMI counts or > 10,000 detected genes were filtered out to remove potential doublets and multiplets. Then scDblFinder (v1.9.3) was applied to further identify potential doublets with its random methods (https://github.com/plger/scDblFinder).

#### Unsupervised cell clustering and annotations

Clustering analysis of the scRNA-seq dataset was performed using the Seurat package (v4.0.5)^42^ and the R program (v4.1.0). UMI count matrices of different samples were merged together for analysis, and then normalized (LogNormalization function, scale.factor = 10,000) and scaled with Seurat. 2000 highly variable features were identified and a PCA matrix with 50 components was constructed. For clustering cells precisely, we applied two round clustering to the dataset. The first-round clustering (resolution = 0.4) identified approximation 5 major cell types including non-immune cells, myeloid cells, T cells, B cells and neutrophils. Clustering results were displayed by UMAP^43^ dimension reduction analysis. A second round of clustering was applied to each major cell type based on a procedure similar to the first round of clustering. For the calculation of cluster markers, we used the sc.tl.rank_genes_groups function of scanpy, and retained the remaining genes for cluster identification after setting certain parameters (threshold > 0.25 and pval_adj < 0.05). Annotation of the final clusters was based on the known cell markers, listed in Supplementary Table S3. Finally, 142,965 identified cells were remaining for downstream analysis. 79 clusters were finally obtained with manual curation, representing different cell subpopulation. Additionally, we found that 17,149 cells in all cells were detected with viral reads (UMI > 0).

#### Temporal distribution of different cell subpopulations

The temporal distribution of selected cell subpopulations was indicated by Ro/e value, which was calculated by the following formula previously described (https://github.com/Japrin/STARTRAC)^44^:

$${Ro}/e=\frac{{observed}}{{expected}}$$

In which the observed variable is the cell number of a given ${{subpopulation}}_{i}$ at a specific ${{timepoint}}_{j}$, and the expected variable is calculated by the following formula, which represents the expected cell number distribution for ${{subpopulation}}_{i}$ at ${{timepoint}}_{j}$:

$${expected}=\frac{{N}_{i}}{{N}_{{all}}}\times {M}_{j}$$

In which ${N}_{{all}}$ is the total cell number of the selected subpopulation (32 subpopulations in Fig.1f), ${N}_{i}$ is the cell number of the given ${{subpopulation}}_{i}$, and ${M}_{j}$ is the cell number of all subpopulations at the specific ${{timepoint}}_{j}$. *Ro/e* > 1, it suggests that cells of the specific subpopulation are more frequently observed than random expectations at the specific timepoint (enrichment). If *Ro/e* < 1, it suggests that cells of the specific subpopulation are observed with less frequency than random expectations at the specific timepoint (depletion). The relevant results were shown in Fig. 1f and Supplementary Table S4.

#### Ligand–receptor analysis by CellChat

Cell types of interest (spatially co-localized) were selected for CellChat analysis. To investigate intercellular interactions among multiple cell types, the CellChat package (v2.1.2) was used to predict active ligand–receptor interactions^26^. We identified 3 groups of cell types for ligand–receptor interactions analysis. Group 1 contained three key cell types in the immunity hubs, alveolar epithelial cells ATI and megakaryocytes. Group 2 contained the union of top 10 cell types with the highest correlation with CD8_T-Cd160 at d2, d5 and d7. Group 3 contained CD8_T-Cd160 and Mac-Slamf9. For each group, we obtained expression lognormalized data matrix as input, and then analyzed with default parameters in https://github.com/sqjin/CellChat. Circle plots were draw with netVisual_circle function. The role of signaling between different cell subpopulations were shown with netAnalysis_signalingRole_heatmap function. Bubble plots between sender and receiver cells were ploted by netVisual_bubble function. The input murine reference ligand–receptor pair list was obtained by CellChatDB.mouse function. We used the netAnalysis_signalingRole_network function in CellChat package to compute importance, and the computed network centrality scores were further visualized by heatmap in Supplementary Fig. S4b.

#### Cell subpopulation similarity analysis between Syrian hamsters and COVID-19 patients

We analyzed snRNA-seq data derived from autopsy lung tissues of COVID-19 patients^28^, to confirm whether subpopulation changes were similar between humans and Syrian hamsters. With homologene R package (v1.4.68.19.3.27), we selected the whole 13,419 homologous genes, including *CD8A/Cd8a*, *CD8B/Cd8b*, *CD160/Cd160*, and *CASP3/Casp3*. We applied a set of signature gene combination to represent the cell type (CD8_T-Cd160: *CD8A*^*+*^*/CD8B*^*+*^*CD160*^*+*^), which performed well in the scRNA-seq data generated by our study. Then we detected cell proportions with expression of signature gene combinations, which were shown in Fig. 3f and Supplementary Fig. S5f.

### Stereo-seq library preparation and sequencing

#### Tissue processing

In the ABSL-3 laboratory, lung tissue frozen sections were adhered to the Stereo-seq chip surface and incubated at 37 °C for 3–5 min. Then, the sections were fixed in methanol and incubated for 40 min at –20 °C before Stereo-seq library preparation. The same cross-linked sections were moved out of the ABSL-3 laboratory and stained with nucleic acid dye (Q10212, Thermo fisher Scientific), and imaging was performed with a Ti-7 Nikon Eclipse microscope prior to in situ capture at the channel of FITC.

#### Library construction and sequencing

In the ABSL-3 laboratory, Stereo-seq library construction was described previously using STOmics_Gene_Expression_kit_S1 (1000028492, MGI)^23^. In brief, RNA released from the tissue was captured by the DNB on the Stereo-chip after tissue sections were permeabilized, followed by reverse transcription. Then the cDNA was released from the stereo-chip for amplification. The concentration of PCR product was quantified by Qubit™ dsDNA Assay Kit (Q32854, Thermo Fisher Scientific). A total of 40–60 ng of DNA was proceeded to fragmentation and amplification. PCR products were purified using the VAHTS^TM^ DNA Clean Beads (0.6× and 0.2×), used for DNB generation, and finally sequenced on MGI DNBSEQ-T10 sequencer.

### Stereo-seq data processing

#### Stereo-seq raw data processing

Fastq files were generated using a MGI DNBSEQ-T10 sequencer. *Mesocricetus auratus* genome (BCM_Maur_2.0) and SARS-CoV-2 genome were integrated as one reference for read mapping by STAR. One base mismatch was allowed to correct sequencing and PCR errors. Mapped reads were counted and annotated using a BGI-developed open-source pipeline SAW (https://github.com/BGIResearch/SAW).

#### Binning data of spatial Stereo-seq data

Because one stereo-chip contained millions of DNBs (diameter: 220 nm), we merged adjacent 80 × 80 DNBs to one bin80 spot (40 μm resolution) as a fundamental unit for downstream analysis, including unsupervised clustering, Redeconve deconvolution calculation and spatial correlation calculation. Additionally, matrices of different spatial Stereo-seq samples were normalized to 1,000,000 counts before analysis.

#### Quality control and batch effect correction of bin80 spatial Stereo-seq data

Stereo-seq data were quality-controlled by filtering low-quality spots for each chip based on the number of detected genes and the total UMI counts in each spot. We got 22,927 bin80 spots per section, 557 genes and 1115 UMI counts per spot of 15 histologically intact bin80 chips after quality control. Then bin80 Stereo-seq data matrices were merged, log-normalized and scaled by the R package Seurat, just like scRNA-seq matrices. We identified 2000 highly variable features across all merged spots and calculated a PCA matrix with 20 components. RunHarmony function in the Harmony package was used to do batch effect correction for different chips^45^.

#### Unsupervised spot clustering and subsets annotations

After unsupervised clustering (resolution = 0.5) calculation, we identified 15 subsets of all selected bin80 spots of 15 selected chips. Clustering results were displayed by their spatial coordination (spatial_x and spatial_y) at the capture chip, which showed the real spatial orientation of spots in tissues. Annotation of the resulting clusters to tissue regions was based on the known markers and the spatial information. Subsets were named by the most enriched cell types or highly expressed genes of clusters (Supplementary Table S2).

#### Spatial transcriptomics deconvolution by Redeconve

We conducted deconvolution analysis of the spatial transcriptomics slides at the bin80 level with the deconvoluting function in the R package Redeconve (v. 0.0.0.9008)^24^, with the average expression profiles of 79 subpopulations identified by our scRNA-seq data as reference. The raw signal from Stereo-seq was first divided into 40 μm × 40 μm grids (i.e., bin80). Genes encoding hemoglobins and rRNAs were excluded from deconvolution analysis because scRNA-seq data excluded red blood cells. The abundance of 79 cell subpopulations within each grid were calculated by using Redeconve with the hyperparameter setting to ten times the suggested value by Redeconve.

#### Spatial correlation calculation between cell subpopulations

After obtaining the cell abundance matrix of each Stereo-seq slide, Pearson’s correlation was used to calculate the spatial correlation between each pair of cell subpopulations (calculated through the coloc.corr function in the R package Redeconve).

#### Cell co-localization

For illustration of the co-localization of three cell subpopulations in spatial data, we used the #FF0000 (CD8_T-Cd160), #0000FF (CD4_T-Tnfrsf4) and #00FF00 (DC-Ccr7-Ido1) channel of RGB color mode to represent each subpopulation. Redeconve abundance of three cell subpopulations higher than 0 was regarded as positive. Finally, the spatial spots were plotted with the converted RGB color. The relevant data were shown in Figs. 3a, 4c and Supplementary Fig. S6b.

#### Spatial module identification for cell subpopulations based on the deconvolution results

After obtaining the spatial correlation matrix (79 cell subpopulations × 79 cell subpopulations) for each Stereo-seq slide, we merged the 9 matrices for each time point into one average matrix. We then performed Louvain clustering based on the 79 × 79 average correlation matrix with the R package NetworkToolbox (v1.4.2)^46^. The clustering results were manually curated to examine the robustness regarding different parameters and across different timepoints. The correlation values larger than 0.25 were reset as 0.25 and those smaller than –0.05 were reset as –0.05 for heatmap drawing in Fig. 2a and Supplementary Fig. S6a. The correlation values larger than 0.3 were reset as 0.3 and those smaller than –0.05 were reset as –0.05 for heatmap drawing in Fig. 4a. Module IDs were manually assigned according to the cell cluster membership. Figure 2c uses the same correlation matrix as Fig. 2a, and the chord diagram was drawn to show the correlation between two cell types higher than 0.05.

### Statistical analysis

Statistics were processed with R statistical software v4.1.2 unless otherwise specified. Two-sided Wilcoxon test, Kruskal–Wallis test and Chi-square test were used as indicated.

## Supplementary information

Supplementary Figures

Supplementary Table S1

Supplementary Table S2

Supplementary Table S3

Supplementary Table S4

Supplementary Table S5

## Data Availability

The data that support the findings of this study have been deposited into CNGB Sequence Archive (CNSA) of China National GeneBank DataBase (CNGBdb) with accession number CNP0002742 and CNP0002978. All other data are included in the article and/or Supplemental information.
