# Supplementary material for: Single-cell spatiotemporal analysis reveals alveolar dendritic cell–T cell immunity hubs defending against pulmonary infection
Source: Cell Discov. 2024 Oct 16;10:103. doi: 10.1038/s41421-024-00733-5 (PMC11484931; doi:10.1038/s41421-024-00733-5)
Supplement: Supplementary file 1 — Supplementary Figures [file 41421_2024_733_MOESM1_ESM.pdf]

# Supplementary Information

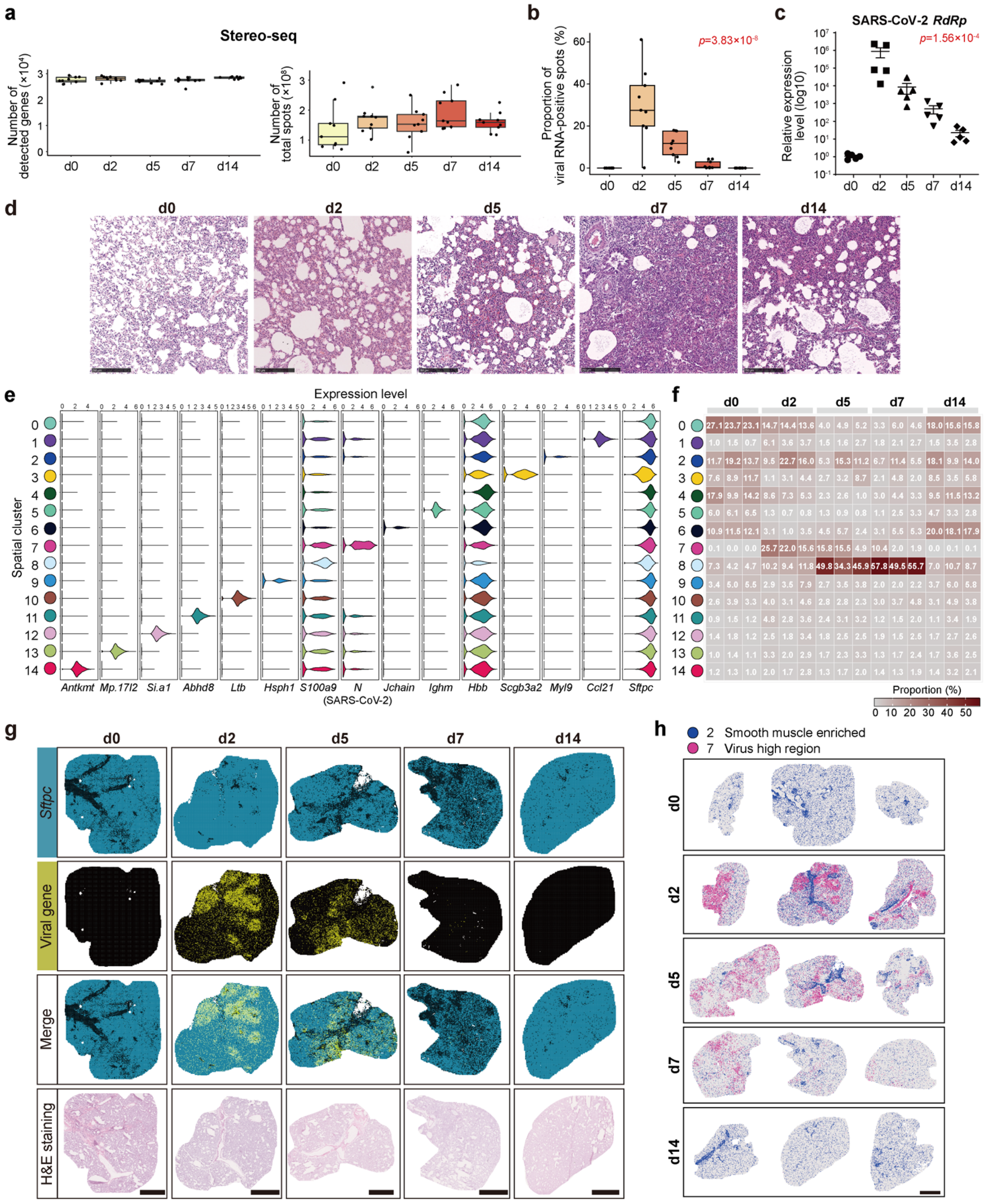


## Supplementary Fig. S1 Spatial transcriptomics of hamster lung sections

**a.** Boxplot showing the quality control of Stereo-seq data, showing total genes detected at the bin80 level and total spot numbers at the bin15 level of 45 spatial transcriptomic sections (nine slides per timepoint). Data are represented as mean±SEM.

**b.** Boxplot showing the proportion of SARS-CoV-2 RNA-positive spots (viral count > 0) of nine lung sections at each timepoint. Center line, median; box bounds, first

and third quartiles; whiskers, 1.5 times the interquartile range. Data are represented as mean±SEM (n=9 slides per timepoint). Kruskal-Wallis test.

**c.** Relative mRNA expression level of SARS-CoV-2 RNA-dependent RNA polymerase (*RdRp*) in the lung lobe of each hamster. Data are represented as mean±SEM (n=5 hamsters per timepoint). Kruskal-Wallis test.

**d.** Representative images of H&E staining showing the histopathological changes of hamster lungs before and after SARS-CoV-2 infection at different timepoints. Scale bar, 250 μm.

**e.** Violin plot showing the expression of selected marker genes to annotate different spatial regions in each spatial cluster.

**f.** Heatmap showing the proportion of each spatial cluster in total spots of each section (three slides per timepoint).

**g.** Spatial transcriptomics data of representative hamster lung tissue slides of each timepoint. Spatial expression patterns of ATII marker gene *Sftpc* and SARS-CoV-2 genes, and H&E staining of the adjacent frozen section were shown. Scale bar, 2 mm.

**h.** The spatial visualization of specific spatial clusters showing the smooth muscle enriched region and virus high region. Scale bar, 2 mm.

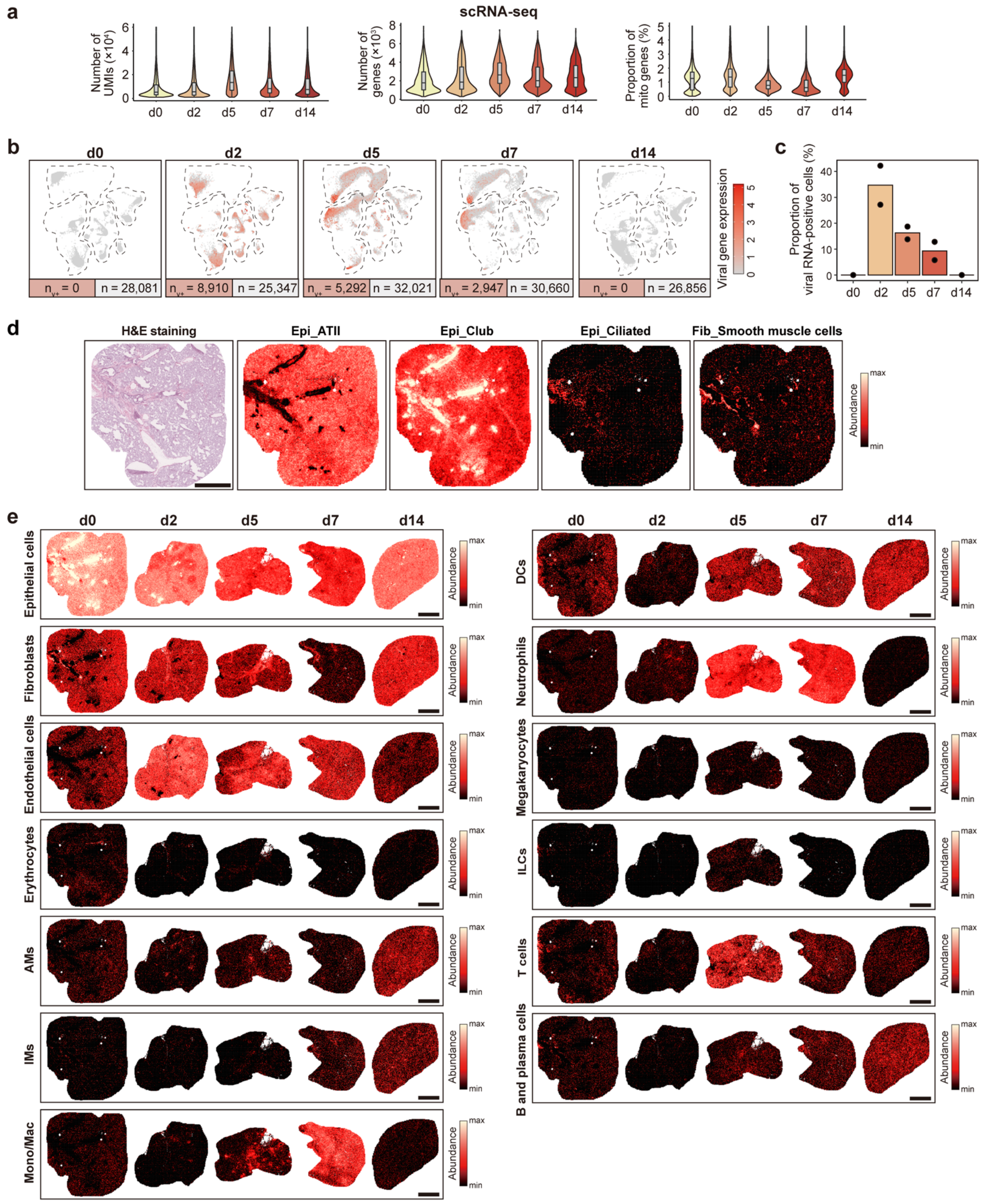


**Supplementary Fig. S2 Quality control of scRNA-seq data and integrative analysis with Stereo-seq**

**a.** Violin plot showing the quality control of scRNA-seq data, showing the distribution of UMI counts per cell, gene numbers per cell, and proportion of mitochondrial genes per cell detected at each timepoint.

**b.** UMAP projection showing the expression of SARS-CoV-2 genes at each timepoint (n, number of total cells; $n_{v+}$, number of virus-positive cells).

**c.** Bar chart showing the proportion of SARS-CoV-2 RNA-positive cells (viral count >

0) of two replicated hamsters at each timepoint.

**d.** Left, H&E staining image of a representative lung slide at d0. Right, spatial distribution pattern of ATII, club cells, ciliated cells and smooth muscle cells. Scale bar, 2 mm.

**e.** Spatial distribution pattern of different cell types at each timepoint. Scale bar, 2 mm.

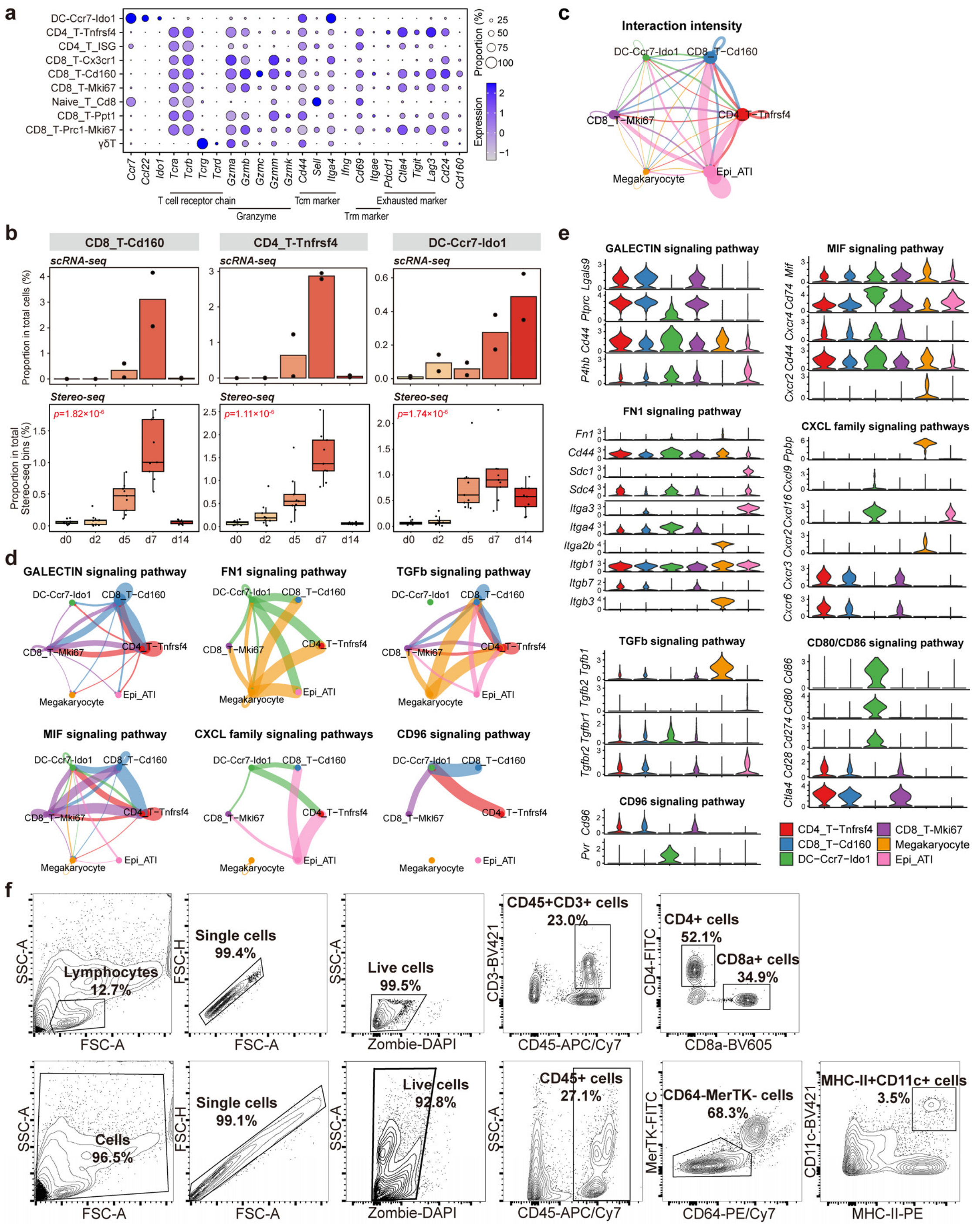

**Supplementary Fig. S3 Intercellular crosstalk pathways among the immunity hubs with alveoli-located cells under physiological conditions**

**a.** Dotplot showing the expression of marker genes in the selected cell subpopulations.

**b.** Top, bar chart showing the proportion of *Ccr7*$^{+}$*Ido1*$^{+}$ DCs, *Cd160*$^{+}$*Cd8*$^{+}$ T cells, and *Tnfrsf4*$^{+}$*Cd4*$^{+}$ T cells in total lung cells detected in two replicated hamsters by scRNA-seq. Bottom, boxplot showing the proportion of these three cell types in total Stereo-seq bin80-bins of nine lung slides at each timepoint. Data are

represented as mean±SEM (n=9 slides per timepoint). Center line, median; box bounds, first and third quartiles; whiskers, 1.5 times the interquartile range. Kruskal-Wallis test.

**c.** Circle plot showing the interaction intensity among three key cell types in the immunity hubs, *Mki67*$^+$*Cd8*$^+$ T cells, megakaryocytes, and ATI, analyzed based on scRNA-seq data. The thickness of the curve indicates the interaction intensity.

**d.** Circle plot showing the intercellular interaction of representative signaling pathways among cell types in the DC-T immunity hubs, *Mki67*$^+$*Cd8*$^+$ T cells, megakaryocytes, and ATI, analyzed based on scRNA-seq data. The thickness of the line indicates the interaction intensity produced by CellChat.

**e.** Violin plot showing the gene expression level of ligand-receptor pairs in the representative signaling pathways in (**d**), analyzed based on scRNA-seq data.

**f.** Gating strategies of flow cytometry analysis detecting CCR7$^+$CD11c$^+$ cells, CD160$^+$CD8$^+$ T cells, and TNFRSF4$^+$CD4$^+$ T cells in the normal lung of C57BL/6J mice (n=5).

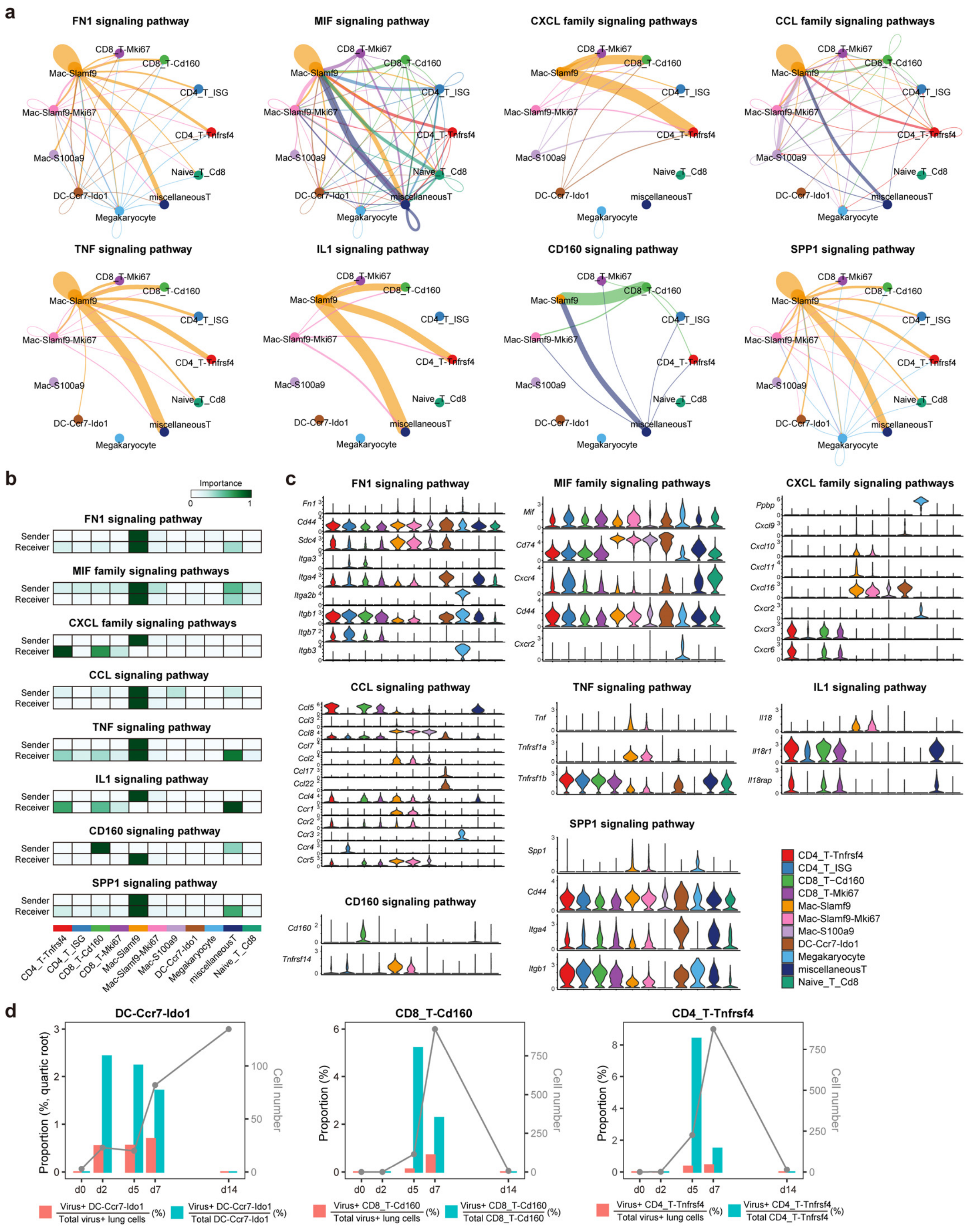

**Supplementary Fig. S4 Intercellular crosstalk pathways in the expanded immunity hubs after SARS-CoV-2 infection**

**a.** Circle plot showing the intercellular interaction of representative signaling pathways among cell types in the expanded immunity hubs. The thickness of the line indicates the interaction intensity produced by CellChat.

**b.** Heatmap showing the relative importance of each cell subset based on computed network centrality measures of the signaling pathways in (**a**). Importance represents the computed network centrality scores produced by CellChat.

**c.** Violin plot showing the gene expression level of ligand-receptor pairs in the signaling pathways in (**a**).

**d.** The number of total *Ccr7*$^{+}$*Ido1*$^{+}$ DCs, *Cd160*$^{+}$*Cd8*$^{+}$ T cells, and *Tnfrsf4*$^{+}$*Cd4*$^{+}$ T cells, the proportion of virus-positive cells and their proportion in total virus-positive cells at each timepoint.

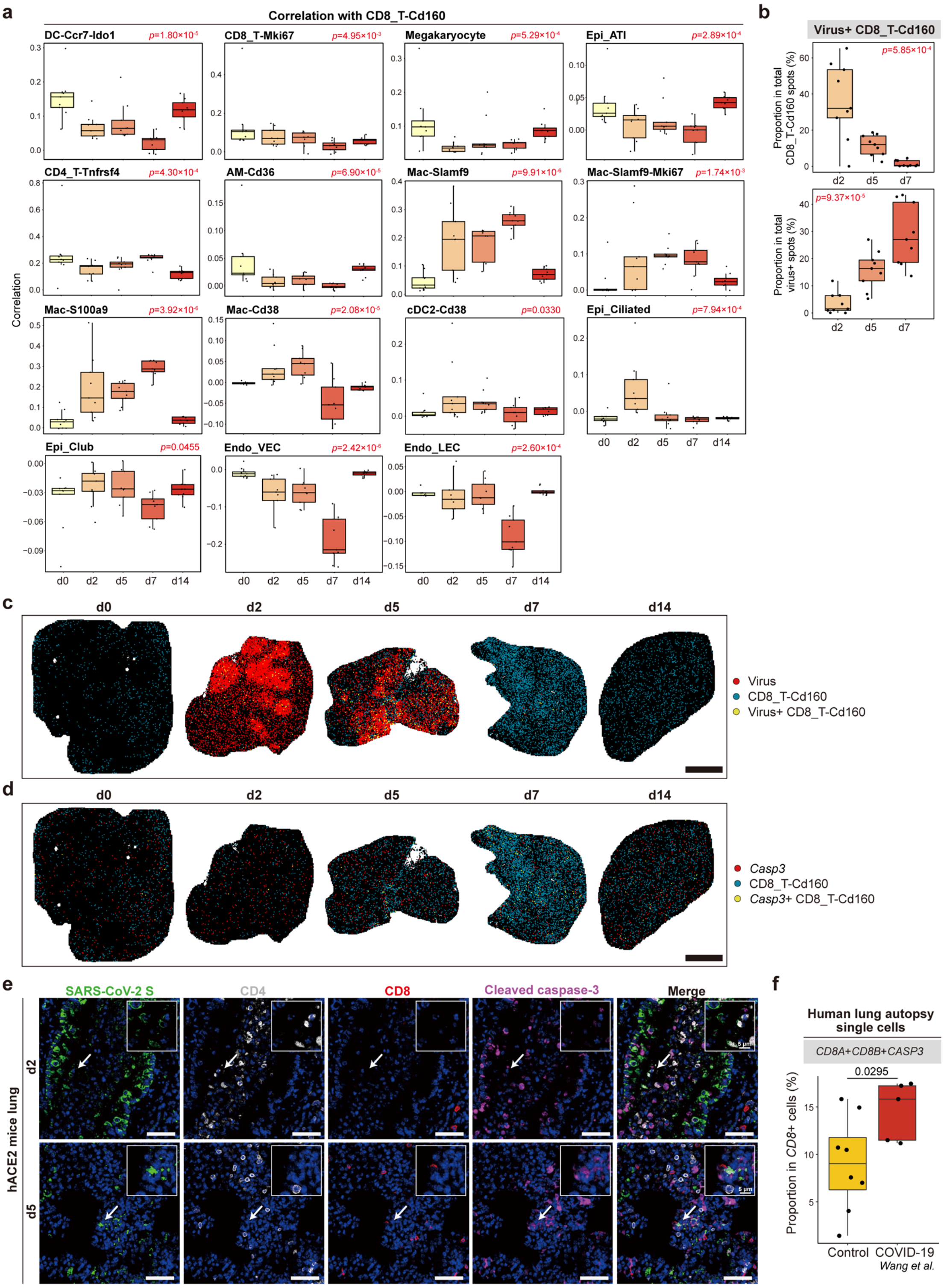


**Supplementary Fig. S5 Signatures of $Cd160^{+}Cd8^{+}$ T cells in response to SARS-CoV-2 infection**

**a.** Boxplot showing the spatial correlation of different cell subpopulations and $Cd160^{+}Cd8^{+}$ T cells of nine lung slides at each timepoint. Center line, median; box

bounds, first and third quartiles; whiskers, 1.5 times the interquartile range. Data are represented as mean±SEM (n=9 slides per timepoint). Kruskal-Wallis test.

**b.** Boxplot showing the spot number of virus-positive *Cd160*$^{+}$*Cd8*$^{+}$ T cells in total *Cd160*$^{+}$*Cd8*$^{+}$ T cells (up) and in total virus-positive bin80-bins (bottom) at each timepoint. Center line, median; box bounds, first and third quartiles; whiskers, 1.5 times the interquartile range. Data are represented as mean±SEM (n=9 slides per timepoint). Kruskal-Wallis test.

**c.** Spatial distribution and co-localization of virus and *Cd160*$^{+}$*Cd8*$^{+}$ T cells, represented by a lung slide at each timepoint. Scale bar, 2 mm.

**d.** Spatial distribution and co-localization of *Casp3* and *Cd160*$^{+}$*Cd8*$^{+}$ T cells, represented by a lung slide at each timepoint. Scale bar, 2 mm.

**e.** Representative images of hACE2 mice lungs showing SARS-CoV-2 S protein, CD4, CD8 and cleaved caspase-3 at d2 and d5 detected by immunofluorescent staining. Co-localizations were indicated by arrows and magnified images. Scale bar, 50 μm.

**f.** Boxplot showing the proportion of *CD8A*$^{+}$*CD8B*$^{+}$*CASP3*$^{+}$ cells in all *CD8*$^{+}$ cells in scRNA-seq data of human lung autopsy samples of control (n=8) and COVID-19 patients (n=5). Data are represented as mean±SEM. Two-sided Wilcoxon test.

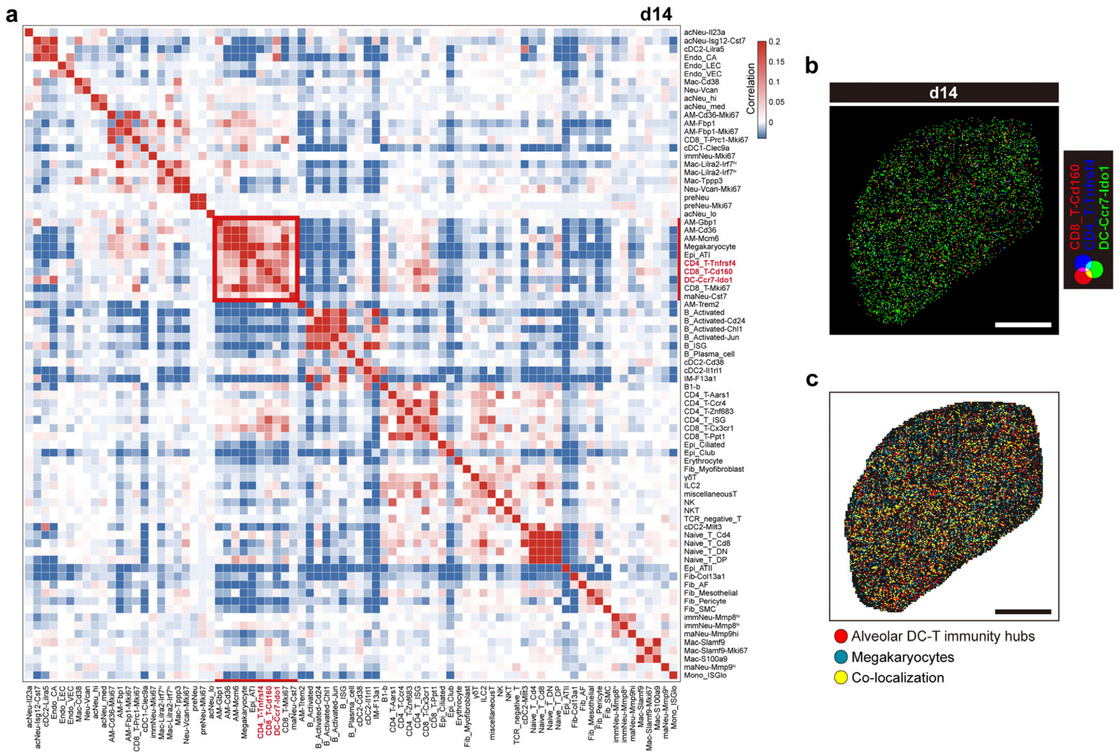


**Supplementary Fig. S6 Restoration of DC-T immunity hubs at the late stage of infection**

**a.** Heatmap of the spatial correlation among 79 cell subpopulations at d14. The module containing *Cd160*$^+$*Cd8*$^+$ T cells was highlighted by the red frame.

**b.** Spatial distribution showing *Ccr7*$^+$*Ido1*$^+$ DCs, *Cd160*$^+$*Cd8*$^+$ T cells, and *Tnfrsf4*$^+$*Cd4*$^+$ T cells in a slide at d14. Co-localization is demonstrated by blending three RGB colors. Scale bar, 2 mm.

**c.** The spatial distribution and the co-localization of key cell types in the DC-T immunity hubs and megakaryocytes in a slide of hamster lung at d14. Scale bar, 2 mm.
